# Assessment of first-touch skills in robotic surgical training using hi-Sim and the hinotori surgical robot system among surgeons and novices

**DOI:** 10.1007/s00423-024-03514-6

**Published:** 2024-11-01

**Authors:** Takeshi Urade, Nobuaki Yamasaki, Munenori Uemura, Junichiro Hirata, Yasuyoshi Okamura, Yuki Mitani, Tatsuya Hattori, Kaito Nanchi, Seiichi Ozawa, Yasuo Chihara, Kiyoyuki Chinzei, Masato Fujisawa, Takumi Fukumoto

**Affiliations:** 1 Department of Surgery, Division of Hepato-Biliary-Pancreatic Surgery, Kobe University Graduate School of Medicine, 7-5-2 Kusunoki-Cho, Chuo-ku, Kobe, 650-0017 Japan; 2 Department of Surgery, Kobe University Graduate School of Medicine, Kobe, Japan; 3 Department of Urology, Kobe University Graduate School of Medicine, Kobe, Japan; 4 Department of Electrical and Electronic Engineering, Kobe University Graduate School of Engineering, Kobe, Japan; 5 International Clinical Cancer Research Center, Kobe University Hospital, Kobe, Japan

**Keywords:** da Vinci, hinotori, hi-Sim, Robot-assisted surgery, Robotic training

## Abstract

**Purpose:**

Surgeons’ adaptability to robotic manipulation remains underexplored. This study evaluated the participants’ first-touch robotic training skills using the hinotori surgical robot system and its simulator (hi-Sim) to assess adaptability.

**Methods:**

We enrolled 11 robotic surgeons (RS), 13 laparoscopic surgeons (LS), and 15 novices (N). After tutorial and training, participants performed pegboard tasks, camera and clutch operations, energizing operations, and suture sponge tasks on hi-Sim. They also completed a suture ligation task using the hinotori surgical robot system on a suture simulator. Median scores and task completion times were compared.

**Results:**

Pegboard task scores were 95.0%, 92.0%, and 91.5% for the RS, LS, and N groups, respectively, with differences between the RS group and LS and N groups. Camera and clutch operation scores were 93.1%, 49.7%, and 89.1%, respectively, showing differences between the RS group and LS and N groups. Energizing operation scores were 90.9%, 85.2%, and 95.0%, respectively, with a significant difference between the LS and N groups. Suture sponge task scores were 90.6%, 43.1%, and 46.2%, respectively, with differences between the RS group and LS and N groups. For the suture ligation task, completion times were 368 s, 666 s, and 1095 s, respectively, indicating differences among groups. Suture scores were 12, 10, and 7 points, respectively, with differences between the RS and N groups.

**Conclusion:**

First-touch simulator-based robotic skills were partially influenced by prior robotic surgical experience, while suturing skills were affected by overall surgical experience. Thus, robotic training programs should be tailored to individual adaptability.

**Supplementary Information:**

The online version contains supplementary material available at 10.1007/s00423-024-03514-6.

## Introduction

Robot-assisted surgery is revolutionizing minimally invasive gastrointestinal procedures [1]. Recently, the introduction of new surgical robots and the inclusion of additional procedures under medical insurance have accelerated the adoption of robotic surgery. Consequently, the number of surgeons training in robotic techniques has surged. The da Vinci Surgical System (Intuitive Surgical, Inc., Sunnyvale, CA, USA) has historically dominated the field, leading to most training reports focusing on the da Vinci and its simulators [2, 3]. In 2020, the hinotori surgical robot system (Medicaroid Corporation, Kobe, Japan) was launched in Japan. This master-slave robotic system, similar to the da Vinci system, features high-precision instruments with 8-axis arms and a three-dimensional (3D) viewer. Unlike the da Vinci Surgical System, the hinotori surgical robot system has demonstrated the capability for telesurgery [4]. Recent studies have highlighted the comparable performance of the hinotori system to the da Vinci surgical system, with its safety validated in clinical studies [5–7]. However, there are no reports on robotic training with the hinotori surgical robot system, and limited knowledge exists regarding its ease of use. Additionally, adaptability of surgeons to robotic manipulation using the hinotori surgical robot system also remains underexplored, despite the evaluation of the transferability of laparoscopic surgical skills to robotic-assisted surgery on the da Vinci Surgical System [8]. Therefore, evaluating the proficiency and adaptability of robotic manipulation using the hinotori surgical robot system and its simulator, hi-Sim, among novices and laparoscopic surgeons is crucial. This study aims to objectively evaluate the proficiency in robotic manipulation using the hinotori system and hi-Sim to determine the adaptability of laparoscopic surgeons and novices during their first exposure.

## Materials and methods

### Participants

We enrolled 11 robotic surgeons (RS group: robotic surgeons with experience in robot-assisted surgery, with a median of 40 cases [range: 1–200]), 13 laparoscopic surgeons (LS group: laparoscopic surgeons with no experience in robot-assisted surgery with a median of 100 cases [range: 100–250] of laparoscopic operations), and 15 novices (N group: medical students with no surgical experience). The baseline demographics of the participants is shown in Table 1. Notably, all robotic surgeons were urologists, and all laparoscopic surgeons were gastroenterologists. Participants in the LS and N groups had no prior exposure to surgical robots or simulators. Data from the RS group served as a benchmark due to their prior experience with robot-assisted surgeries. Informed consent was obtained from all participants.

**Table 1:** Baseline demographics of participants in the robot surgeons, laparoscopic surgeons, and novices groups

	Factor	Value		
Robot surgeons (*n* = 11)	Age (years) §	47 (35–51)		
	Gender			
	Male	10 (91)		
	Female	1 (9)		
	Vision correction	8 (73)		
	Experience of regularly playing video games			
	Yes	3 (27)		
	No	8 (73)		
	daVinci console surgeon	11 (100)		
	hionotori cockpit surgeon	8 (73)		
	Average number as operator			
	Robot surgery §	40 (30–100)		
	Laparoscopic surgery §	100 (30–100)		
	Open surgery §	100 (30–200)		
Laparoscopic surgeons (*n* = 13)	Age (years) §	36 (32–40)		
	Gender			
	Male	13 (100)		
	Vision correction	9 (69)		
	Experience of regularly playing video games			
	Yes	7 (54)		
	No	6 (46)		
	Average number as operator			
	Laparoscopic surgery §	100 (100–200)		
	Open surgery §	200 (75–300)		
Novices (*n* = 15)	Age (years) §	24 (23–24)		
	Gender			
	Male	12 (80)		
	Female	3 (20)		
	Vision correction	12 (80)		
	Experience of regularly playing video games			
	Yes	7 (47)		
	No	8 (53)		
	Training level			
	5th-year medical student	8 (53)		
	6th-year medical student	7 (47)		

Values in parentheses are percentages unless indicated otherwise; §values are median (interquartile range)

## Study protocol

**Fig. 1:**
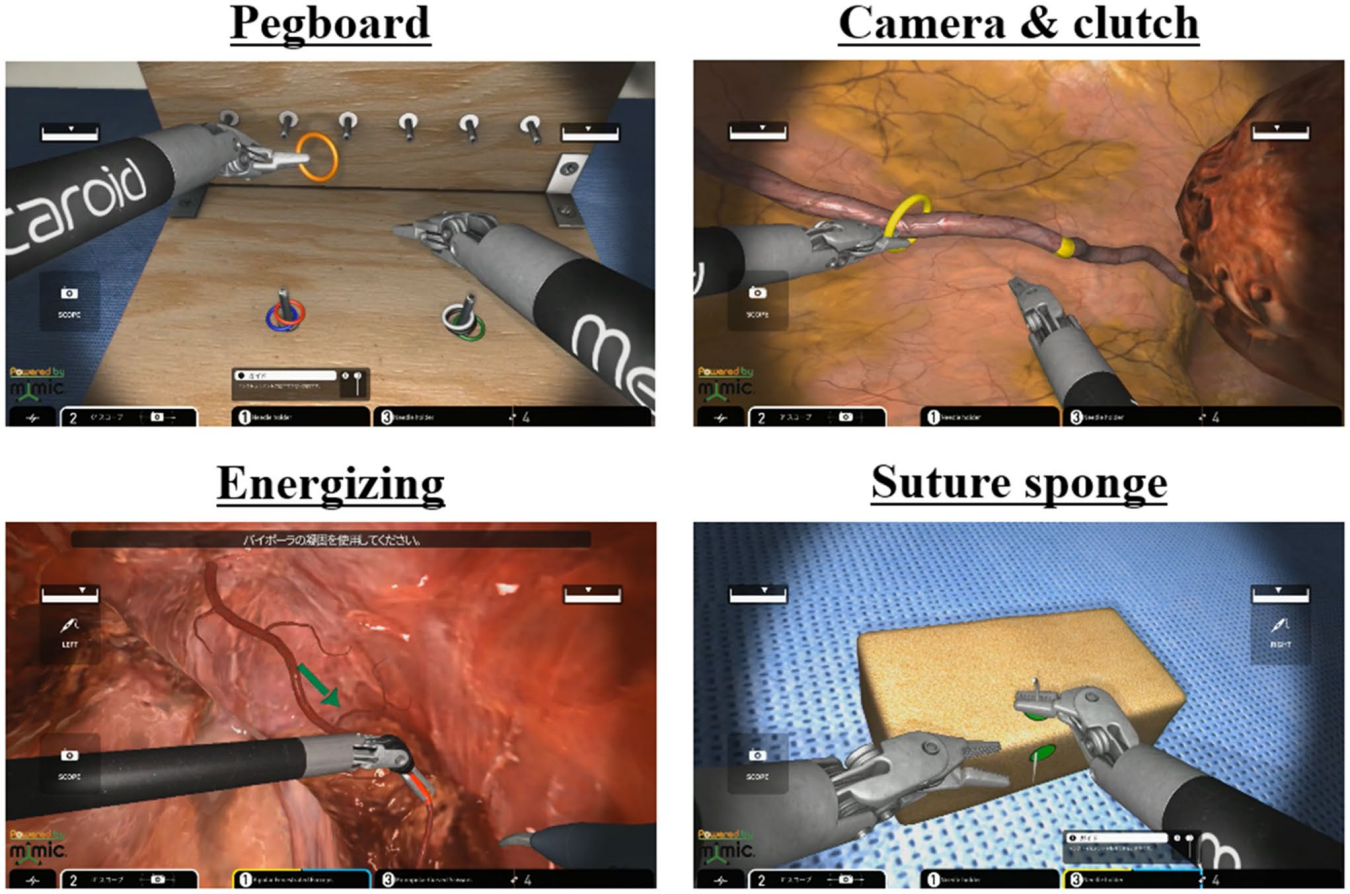
Four simulator tasks on the hi-Sim

**Fig. 2:**
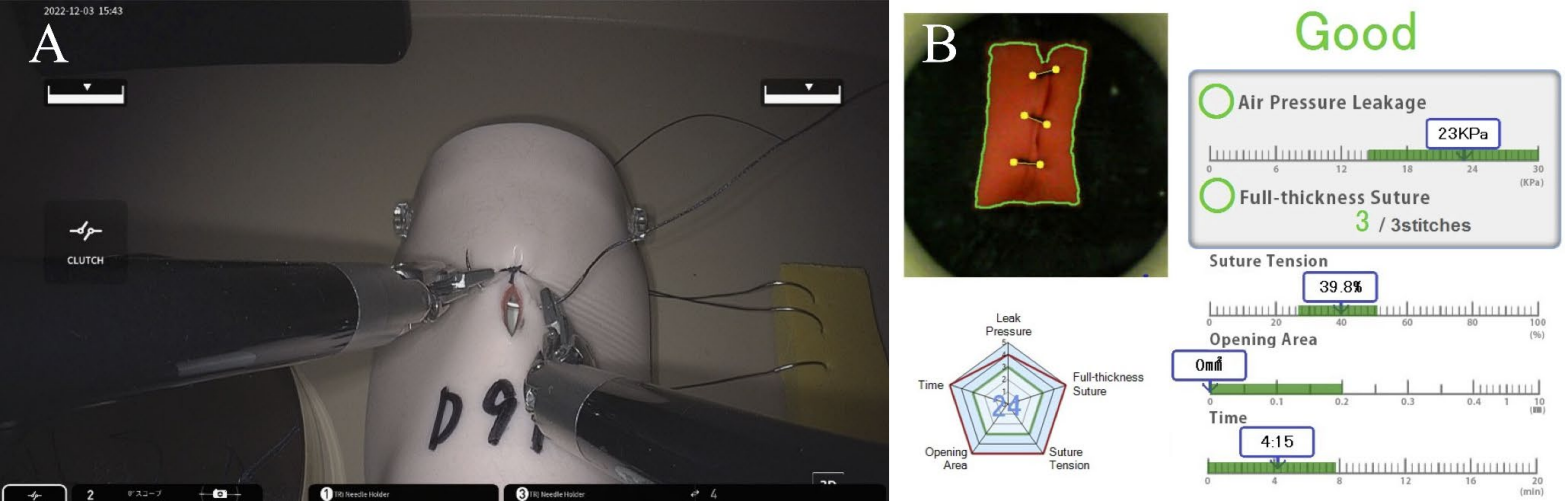
(**A**) Suture ligation task using the hinotori surgical robot system. (**B**) Scoring system on the A-LAP mini

The hinotori surgical robot system and its simulator, hi-Sim, are used at the International Clinical Cancer Research Center, Kobe University Hospital. Participants underwent a tutorial followed by a 15-minute training session with a pick-and-place task on hi-Sim. Subsequently, they performed four simulator tasks on the hi-Sim: pegboard, camera and clutch operation, energizing operation, and suture sponge (Fig. 1). Additionally, they completed a suture ligation task using the hinotori surgical robot system on a suture simulator (A-LAP mini; KYOTO KAGAKU, Kyoto, Japan) (Fig. 2). Brief instructions were provided for each task. The suture ligation task involved wound closure using three sutures, with an assistant cutting the thread after each suture.

## Performance metrics

Performance on the simulator tasks was evaluated based on various metrics using the built-in scoring system in the hi-Sim software. The exercise metrics are defined as follows:

1. Time to complete exercise: Total time the user spends on the exercise.
2. Economy of motion: Total distance traveled by all instruments.
3. Master workspace range: The larger of the two radii of motion of the user’s working volume on master grips.
4. Instrument collisions: Total number of instrument-on-instrument collisions exceeding a minimum force threshold.
5. Excessive instrument force: Total time an excessive instrument force is applied above a prescribed threshold force. Forces on an instrument can arise from collisions with each other and from actions such as tissue retraction, driving a needle, pulling on sutures, etc.
6. Instrument out of view: Total distance traveled by instruments outside the user’s field of view.
7. Drops: The number of times any object is dropped in an inappropriate region of the scene.
8. Misapplied energy time: Total time an incorrect energy is applied to a target or energy is enabled while not touching a target.
9. Missed targets: The number of missed targets.

These definitions were sourced from Surgical Science Sweden AB. with their permission. However, the equations used to assess robotic surgical skills were confidential. Completion time was recorded for the suture ligation task. Additional metrics such as air pressure leakage, full-thickness sutures, suture tension, and opening area of the sutured artificial intestinal models were measured in a pressurized chamber [9]. The A-LAP mini software was employed to score all metrics, with a maximum score of 25 points for each.

### Statistical analysis

All data were analyzed using JMP ver. 16.0 software (SAS Institute Inc., Cary, NC, USA). Performance scores among the groups were compared using the Steel–Dwass test. Statistical significance was set at *p* < 0.05.

## Results

**Fig. 3:**
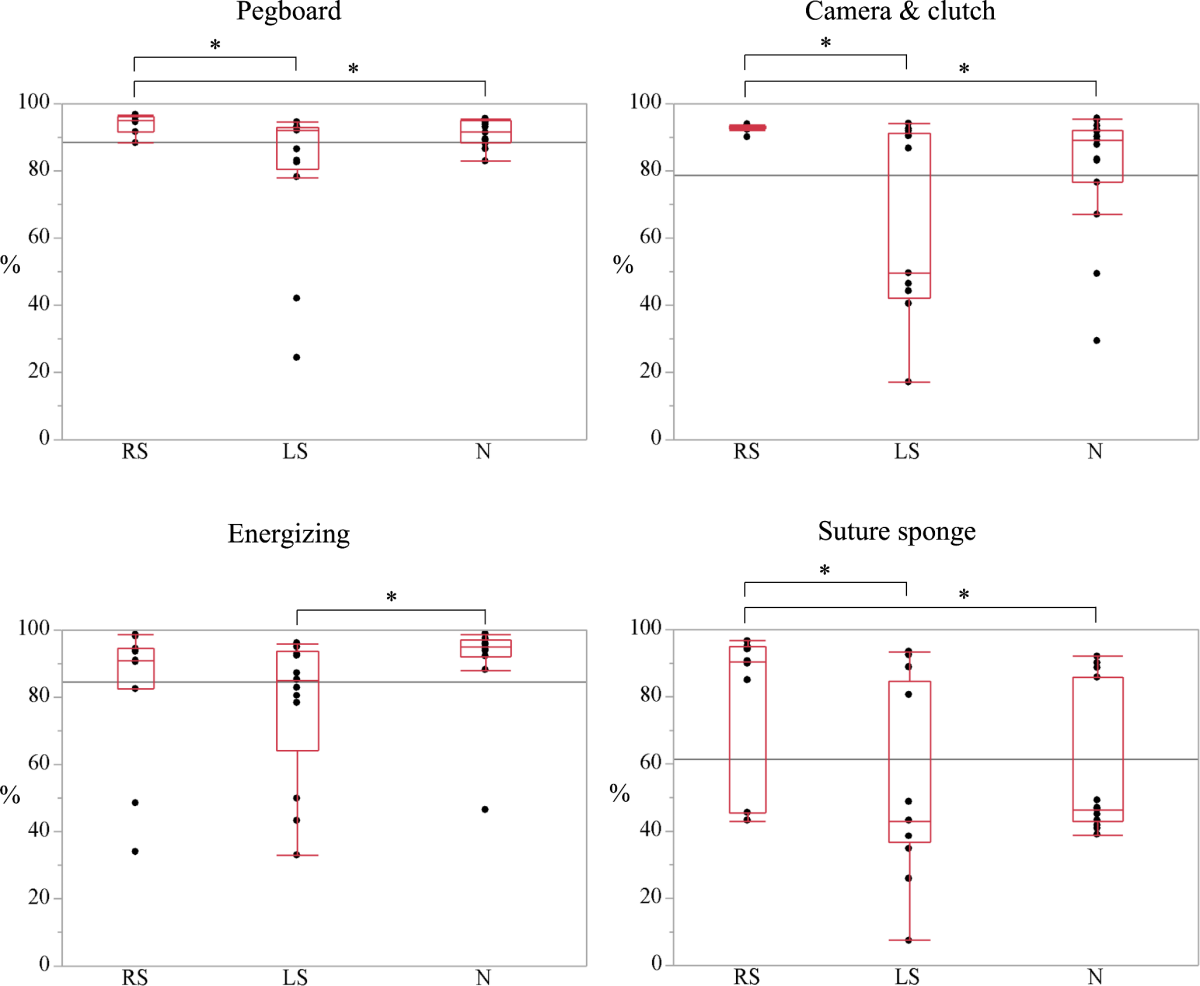
Scores of the four tasks on the hi-Sim. *p < 0.05 between groups

Scores of the simulator skills on hi-Sim are shown in Fig. 3 and Table S1. The pegboard scores were 95.0%, 92.0%, and 91.5% for RS, LS, and N groups, respectively, with significant differences observed between the RS and LS groups (*p* = 0.012) and between the RS and the N groups (*p* = 0.027). The camera and clutch operations scores were 93.1%, 49.7%, and 89.1%, respectively, showing significant differences between the RS and the LS groups (*p* = 0.004) and between the RS and the N groups (*p* = 0.031). The energizing operation scores were 90.9%, 85.2%, and 95.0%, respectively, indicating a significant difference between the LS and the N groups (*p* = 0.021). The suture sponge scores were 90.6%, 43.1%, and 46.2%, respectively, with significant differences between the RS and the LS groups (*p* = 0.023) and between the RS and the N groups (*p* = 0.044).

**Fig. 4:**
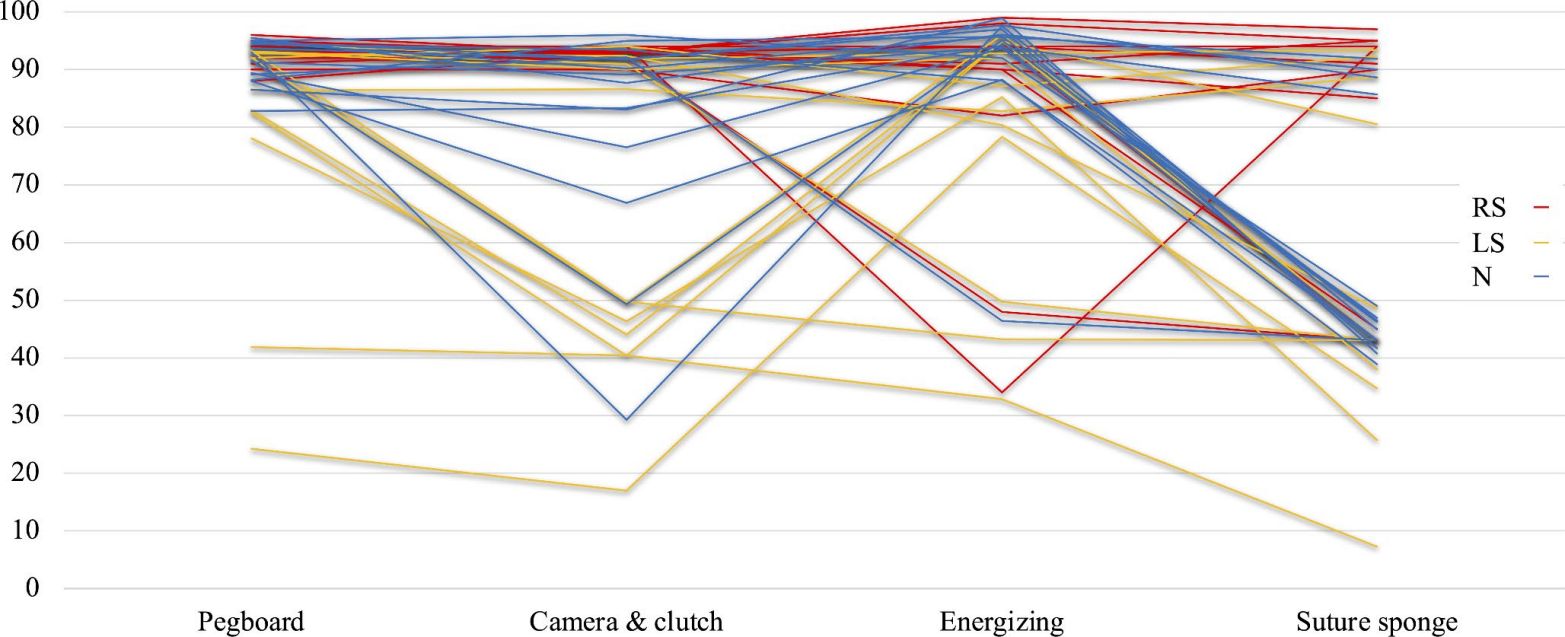
Scores of each participant on the hi-Sim tasks

Figure 4 depicts individual scores for each participant on the hi-Sim tasks. Only one participant in the RS group (9.1%) had multiple low scores below 80%, compared to five participants (33.3%) in the N group and seven participants (53.8%) in the LS group. Subgroup analyses for each task are detailed in Tables S2–S5.

**Fig. 5:**
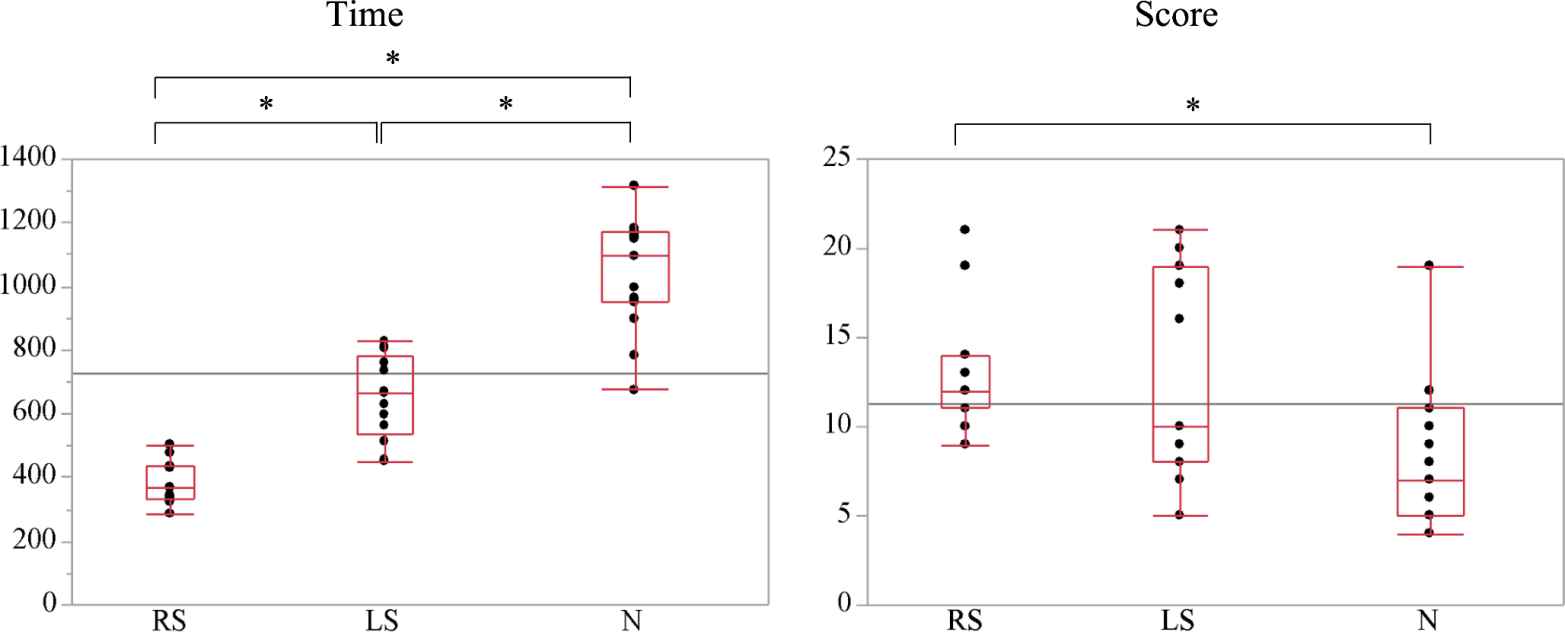
Time and scores of the suture ligation task using the hinotori surgical robot system. *p < 0.05 between groups

The scores for the suture ligation task on the hinotori surgical robot system are shown in Fig. 5. Suture times were 368 s, 666 s, and 1095 s, respectively, with significant differences between the RS and LS groups (*p* < 0.001), RS and N groups (*p* < 0.001), and LS and N groups (*p* < 0.001). Median suture scores were 12, 10, and 7 points, respectively, with significant differences between the RS and N groups (*p* = 0.006).

## Discussion

This study represents the first investigation into robotic training using the hinotori surgical robot system and hi-Sim, contrasting with numerous studies focusing on the da Vinci system and its simulators. We believe that our findings can be extended to other master–slave robot systems.

In the simulator tasks on hi-Sim, the RS group significantly outperformed the LS and N groups in the pegboard, camera clutch, and suture sponge tasks. However, metrics for automatically calculating the performance score metrics should be taken into consideration. Notably, the RS group exhibited shorter performance times and fewer instances of instrument interference on the pegboard task compared to the LS and N groups (Table S2). Although differences in camera and clutch tasks were minimal, the RS group still displayed superior results overall (Table S3). Generally, the RS group demonstrated shorter performance time, better economy of motion, fewer instrument collisions, and fewer missed targets compared to the LS and N groups (Table S5), indicating that robotic surgical experience positively influences simulator skills. Interestingly, the N group had significantly better energizing scores than the LS group and achieved the highest median score among the three groups. Furthermore, they had the shortest misapplied energy time (Table S4). In other words, they made fewer mistakes when using foot pedals for monopolar or bipolar coagulation. The energizing task differs from others as it requires additional foot pedal operations, which may lead to errors. The differences in performance appear independent of factors such as gender, vision correction, or video game experience, suggesting that age may play a significant role. Starting robotic training at a younger age could therefore be beneficial. Reducing errors among laparoscopic and robotic surgeons is the next challenge to address. The LS and N groups showed minimal significant differences in the simulator tasks. In the LS group, the adaptability to perform the simulator tasks at the first exposure did not necessarily correlate with their experience in laparoscopic surgery. This suggests that prior laparoscopic surgical experience may not be a prerequisite for performing robotic simulator tasks, aligning with the results of previous studies [10–13]. As shown in Fig. 4, the LS group had more participants with multiple low scores under 80% compared to the other groups. This suggests that these surgeons struggled to adapt to the unfamiliar robotic manipulations during their first exposure. Several factors may explain these results. First, the method of moving the forceps differs between laparoscopic and robotic surgery. In laparoscopic surgery, the movement of the forceps’ tip within the abdominal cavity mirrors the external movement both vertically and horizontally. Conversely, robotic surgery allows for more intuitive manipulation of the forceps’ tip, requiring laparoscopic surgeons to adjust to robot-specific movements. Second, younger individuals may be more adept at adapting to robotic movements due to their familiarity with computer interfaces, 3D movies, and virtual reality systems, including video games, as previously reported [13, 14]. However, caution is warranted in generalizing these results due to the small sample size and participant characteristics, as some studies have reported a positive effect of prior laparoscopic training on initial robotic simulator scores [10, 12, 15].

In the suture ligation task using the hinotori surgical robot system, the RS group completed the task significantly faster than the LS group, which, in turn, was faster than the N group. Additionally, while the RS and LS groups performed similarly in suturing scores, the RS group significantly outperformed the N group. Thus, we speculate that first-touch suture ligation skills are influenced by both laparoscopic and robotic surgical experience. Various reports have indicated that laparoscopic training positively impacts advanced surgical skills such as intracorporeal knot-tying and suturing [16, 17]. In this study, laparoscopic skills appear transferrable to robotic surgery, particularly for complex surgical techniques. However, there are unique aspects of robotic intracorporeal suture ligation that differ from those of laparoscopic ligation, such as the inability to perceive the depth of needle advancement or gastrointestinal tract tissue thickness and the potential for thread tearing due to the lack of tactile feedback. Therefore, laparoscopic surgeons must acquire specific robotic techniques to compensate for this lack of tactile sensation.

Despite its strengths, this study had some limitations. First, all the robotic surgeons were urologists, and all the laparoscopic surgeons were general surgeons. The differences in surgical procedures between urology and general surgery complicate the direct application of these results to general robotic surgeons. Additionally, the varying surgical experiences among the surgeons might have influenced the results. Second, first-touch skills were evaluated using only the hinotori surgical robot system. Applying these results directly to the da Vinci surgical system or other robots may be challenging despite the similar basic structures of the hinotori and da Vinci systems. A comparative study between hinotori and da Vinci’s training skills is required. Third, the learning curve for operating the hinotori system should be elucidated, as it was not evaluated in this study. Lastly, since only the hinotori surgical robot system is currently approved for use in Japan, the enrollment of only Japanese surgeons and novices might have influenced the results due to differences in surgical training and education.

The future of robotic surgical training lies in the strategic integration of log analysis and artificial intelligence technologies. Harnessing these tools can enhance the precision, efficiency, and effectiveness of surgical training, ultimately improving patient outcomes and advancing the field of robotic surgery. Further studies are required to optimize robotic training for users of surgical systems.

## Conclusion

The first-touch simulator skills were partially influenced by previous robotic surgical experience, and the actual suturing skills were influenced by overall surgical experience. Consequently, robotic training should be tailored to individual adaptability to robotic manipulation during the initial exposure and to individual surgical experience.

## Electronic supplementary material

Below is the link to the electronic supplementary material.

Supplementary Material 1

Supplementary Material 2

Supplementary Material 3

Supplementary Material 4

Supplementary Material 5

## Data Availability

No datasets were generated or analysed during the current study.
