# Supplementary material for: Assessment of first-touch skills in robotic surgical training using hi-Sim and the hinotori surgical robot system among surgeons and novices
Source: Langenbecks Arch Surg. 2024 Nov 1;409(1):332. doi: 10.1007/s00423-024-03514-6 (PMC11527936; doi:10.1007/s00423-024-03514-6)
Supplement: Supplementary file 1 — Supplementary Material 1 [file 423_2024_3514_MOESM1_ESM.docx]

| **Table S1.** Result of each task in the hi-Sim and the hinotori surgical robot system. | | | | | | | |
| --- | --- | --- | --- | --- | --- | --- | --- |
|  | RS | LS | N |  | *P value* | | |
|  |  |  |  |  | RS vs. LS | RS vs. N | LS vs. N |
| hi-Sim |  |  |  |  |  |  |  |
| Pegboard (%) | 95.0 (91.5–96.2) | 92.0 (80.3–93.0) | 91.5 (88.2–94.8) |  | 0.012 | 0.027 | 0.260 |
| Camera & clutch (%) | 93.1 (92.5–93.5) | 49.7 (42.3–91.4) | 89.1 (76.5–92.1) |  | 0.004 | 0.031 | 0.375 |
| Energizing (%) | 90.9 (82.4–94.4) | 85.2 (64.1–93.9) | 95.0 (92.2–97.0) |  | 0.623 | 0.354 | 0.021 |
| Suture sponge (%) | 90.6 (45.4–94.9) | 43.1 (36.6–84.6) | 46.2 (43.1–85.7) |  | 0.023 | 0.044 | 0.766 |
| hinotori |  |  |  |  |  |  |  |
| Suture time (sec) | 368 (335–435) | 666 (538–783) | 1095 (950–1174) |  | < 0.001 | < 0.001 | < 0.001 |
| Suture score (points) | 12 (11–14) | 10 (8–19) | 7 (5–11) |  | 0.830 | 0.006 | 0.084 |
| Values are median (interquartile range). | | | | | | | |
