# Supplementary material for: Assessment of first-touch skills in robotic surgical training using hi-Sim and the hinotori surgical robot system among surgeons and novices
Source: Langenbecks Arch Surg. 2024 Nov 1;409(1):332. doi: 10.1007/s00423-024-03514-6 (PMC11527936; doi:10.1007/s00423-024-03514-6)
Supplement: Supplementary file 2 — Supplementary Material 2 [file 423_2024_3514_MOESM2_ESM.docx]

| **Table S2.** Comparison of the task evaluation element for Pegboard in the hi-Sim. | | | | | | | |
| --- | --- | --- | --- | --- | --- | --- | --- |
| **Pegboard** | RS | LS | N |  | *P value* | | |
|  |  |  |  |  | RS vs. LS | RS vs. N | LS vs. N |
| Time to complete exercise (sec) | 107 (94–124) | 189 (134–381) | 183 (142–212) |  | 0.002 | 0.005 | 0.769 |
| Economy of motion (cm) | 266 (245–295) | 340 (274–570) | 268 (243–341) |  | 0.021 | 0.930 | 0.055 |
| Master workspace range (cm) | 9.5 (7.5–10.3) | 10.6 (7.9–13.5) | 11.4 (10.4–12.6) |  | 0.443 | 0.006 | 0.795 |
| Instrument collisions (times) | 0 (0–0) | 3 (2–9) | 2 (0–4) |  | 0.001 | 0.017 | 0.282 |
| Excessive instrument force (sec) | 0 (0–0) | 0 (0–0.9) | 0 (0–0) |  | 0.589 | 0.926 | 0.728 |
| Instrument out of view (cm) | 0 (0–3.1) | 0.2 (0–1.1) | 0 (0–0.4) |  | 0.681 | 0.904 | 0.437 |
| Drops (times) | 0 (0–0) | 1 (1–1) | 1 (0–1) |  | 0.004 | 0.059 | 0.374 |
| Values are median (interquartile range). | | | | | | | |
