# Supplementary material for: Assessment of first-touch skills in robotic surgical training using hi-Sim and the hinotori surgical robot system among surgeons and novices
Source: Langenbecks Arch Surg. 2024 Nov 1;409(1):332. doi: 10.1007/s00423-024-03514-6 (PMC11527936; doi:10.1007/s00423-024-03514-6)
Supplement: Supplementary file 3 — Supplementary Material 3 [file 423_2024_3514_MOESM3_ESM.docx]

| **Table S3.** Comparison of the task evaluation elements for Camera & clutch in the hi-Sim. | | | | | | | |
| --- | --- | --- | --- | --- | --- | --- | --- |
| **Camera & Clutch** | RS | LS | N |  | *P value* | | |
|  |  |  |  |  | RS vs. LS | RS vs. N | LS vs. N |
| Time to complete exercise (sec) | 98 (75–114) | 129 (98–168) | 128(96–174) |  | 0.106 | 0.058 | 0.982 |
| Economy of motion (cm) | 98 (78–132) | 114 (85–190) | 93 (83–139) |  | 0.550 | 0.887 | 0.598 |
| Master workspace range (cm) | 9.1 (5.8–10.1) | 10.7 (7.8–14.1) | 11.0 (9.4–14.3) |  | 0.377 | 0.095 | 0.919 |
| Instrument collisions (times) | 0 (0–2) | 1 (0–3) | 1 (0–5) |  | 0.335 | 0.317 | 1.000 |
| Excessive instrument force (sec) | 0 (0–0) | 0 (0–1.9) | 0 (0–0) |  | 0.134 | 0.293 | 0.748 |
| Instrument out of view (cm) | 0 (0–0) | 2 (0–7.1) | 0.8 (0–3.0) |  | 0.033 | 0.083 | 0.617 |
| Values are median (interquartile range). | | | | | | | |
