# Supplementary material for: Assessment of first-touch skills in robotic surgical training using hi-Sim and the hinotori surgical robot system among surgeons and novices
Source: Langenbecks Arch Surg. 2024 Nov 1;409(1):332. doi: 10.1007/s00423-024-03514-6 (PMC11527936; doi:10.1007/s00423-024-03514-6)
Supplement: Supplementary file 4 — Supplementary Material 4 [file 423_2024_3514_MOESM4_ESM.docx]

| **Table S4.** Comparison of the task evaluation element for Energizing in the hi-Sim. | | | | | | | |
| --- | --- | --- | --- | --- | --- | --- | --- |
| **Energizing** | RS | LS | N |  | *P value* | | |
|  |  |  |  |  | RS vs. LS | RS vs. N | LS vs. N |
| Time to complete exercise (sec) | 88 (81–105) | 142 (107–178) | 125 (96–141) |  | 0.011 | 0.066 | 0.401 |
| Economy of motion (cm) | 125 (108–150) | 149 (117–200) | 120 (102–138) |  | 0.261 | 0.586 | 0.034 |
| Master workspace range (cm) | 7.1 (5.8–9.4) | 9.3 (6.4–11.4) | 10.3 (9.0–13.3) |  | 0.478 | 0.022 | 0.468 |
| Instrument collisions (times) | 0 (0–0) | 0 (0–0) | 0 (0–1) |  | 0.413 | 0.102 | 0.594 |
| Excessive instrument force (sec) | 0 (0–1.9) | 0 (0–4.1) | 0 (0–0) |  | 0.845 | 0.099 | 0.029 |
| Instrument out of view (cm) | 0 (0–1.1) | 0 (0–3.8) | 0 (0–0) |  | 0.980 | 0.343 | 0.241 |
| Misapplied energy time (sec) | 7.5 (2.7–9.7) | 6.5 (3.9–10.8) | 1.0 (0.5–3.0) |  | 1.000 | 0.004 | < 0.001 |
| Values are median (interquartile range). | | | | | | | |
