# Supplementary material for: Assessment of first-touch skills in robotic surgical training using hi-Sim and the hinotori surgical robot system among surgeons and novices
Source: Langenbecks Arch Surg. 2024 Nov 1;409(1):332. doi: 10.1007/s00423-024-03514-6 (PMC11527936; doi:10.1007/s00423-024-03514-6)
Supplement: Supplementary file 5 — Supplementary Material 5 [file 423_2024_3514_MOESM5_ESM.docx]

| **Table S5.** Comparison of the task evaluation element for Suture sponge in the hi-Sim. | | | | | | | |
| --- | --- | --- | --- | --- | --- | --- | --- |
| **Suture sponge** | RS | LS | N |  | *P value* | | |
|  |  |  |  |  | RS vs. LS | RS vs. N | LS vs. N |
| Time to complete exercise (sec) | 152 (124–192) | 364 (283–548) | 348 (291–375) |  | < 0.001 | < 0.001 | 0.769 |
| Economy of motion (cm) | 226 (199–286) | 513 (364–706) | 349 (336–436) |  | < 0.001 | < 0.001 | 0.142 |
| Master workspace range (cm) | 6.6 (5.4–8.2) | 7.9 (5.7–10.4) | 8.6 (7.2–9.5) |  | 0.800 | 0.084 | 0.713 |
| Instrument collisions (times) | 3 (0–7) | 16 (5–31) | 16 (6–22) |  | 0.015 | 0.015 | 0.965 |
| Excessive instrument force (sec) | 0.5 (0–3.1) | 4.7 (0.5–11.0) | 2.7 (0–9.4) |  | 0.133 | 0.770 | 0.384 |
| Instrument out of view (cm) | 3.5 (0.6–5.7) | 5.2 (2.3–31.8) | 2.0 (0–5.3) |  | 0.476 | 0.834 | 0.168 |
| Drops (times) | 0 (0–0) | 0 (0–2.5) | 0 (0–2) |  | 0.281 | 0.526 | 0.705 |
| Missed target (times) | 1 (0–3) | 6 (5–17) | 6 (3–12) |  | 0.004 | 0.008 | 0.959 |
| Values are median (interquartile range). | | | | | | | |
